# Impact of the Front-of-Pack Label Nutri-Score on the Nutritional Quality of Food Choices in a Quasi-Experimental Trial in Catering

**DOI:** 10.3390/nu13124530

**Published:** 2021-12-17

**Authors:** Chantal Julia, Nathalie Arnault, Cédric Agaësse, Morgane Fialon, Mélanie Deschasaux-Tanguy, Valentina A. Andreeva, Léopold K. Fezeu, Emmanuelle Kesse-Guyot, Mathilde Touvier, Pilar Galan, Serge Hercberg

**Affiliations:** 1 Nutritional Epidemiology Research Team (EREN), Sorbonne Paris Cité Epidemiology and Statistics Research Center (CRESS), Inserm U1153, Inrae U1125, Cnam, Université Sorbonne Paris Nord University, 93017 Bobigny, France; n.arnault@eren.smbh.univ-paris13.fr (N.A.); c.agaesse@eren.smbh.univ-paris13.fr (C.A.); m.fialon@eren.smbh.univ-paris13.fr (M.F.); m.deschasaux@eren.smbh.univ-paris13.fr (M.D.-T.); v.andreeva@eren.smbh.univ-paris13.fr (V.A.A.); l.fezeu@eren.smbh.univ-paris13.fr (L.K.F.); e.kesse@eren.smbh.univ-paris13.fr (E.K.-G.); m.touvier@eren.smbh.univ-paris13.fr (M.T.); p.galan@eren.smbh.univ-paris13.fr (P.G.); s.hercberg@eren.smbh.univ-paris13.fr (S.H.); 2 Public Health Department, Paris-Seine-Saint-Denis University Hospitals (AP-HP), 93017 Bobigny, France

**Keywords:** front-of-pack labelling, dietary intake, quasi-experimental trial, catering

## Abstract

The front-of-pack labelling Nutri-Score has recently been implemented as a policy measure to improve the healthiness of food choices. The aim of this study was to investigate the impact of the Nutri-Score label in catering. A quasi-experimental trial was conducted in France between 16 December 2019 and 13 March 2020 in two staff restaurants (one intervention and one control site) from the same company. After a control period of seven weeks, the Nutri-Score label was affixed on all proposed products in the intervention site. Overall effects of the intervention were investigated using a difference in difference approach with generalised linear models. Over the 13 weeks of the study, 2063 participants who frequented the restaurant cafeteria at least once were included (1268 and 795 in the intervention and control site, respectively), representing 36,114 meals. Overall, the intervention led to a significant improvement in the nutritional quality of meals (*p* = 0.008) and a significant reduction in the intake of calories, sugars and saturated fat (*p* < 0.0001). Mixed effects models showed a qualitative improvement of food choices initially, and an adaptation of the quantities consumed over time, suggesting for the first time longer-term effects of the label on dietary behaviour.

## 1. Introduction

Policy approaches in the area of nutrition have recently put forward front-of-pack nutrition labels as efficient tools to help consumers make healthier choices at the point of purchase, and contribute to the prevention of nutrition-related diseases [1,2]. Front-of-pack labels (FOPLs) provide simplified and ready-to-use nutritional information in addition to the nutritional declaration on food packages, to facilitate comparisons among products in the constrained environment of food purchases [3,4]. Interpretive FOPLs are considered by the World Health Organization as one of the ‘best buys’ policies to tackle nutrition-related diseases [5].

A growing number of countries have implemented either voluntary or mandatory systems, with a large variability in the formats adopted depending on the geographical area [6]. In the European region, seven countries have now adopted the Nutri-Score as a voluntary system—consistently with the EU regulation excluding mandatory measures—including Belgium, France, Germany, Luxemburg, the Netherlands, Spain and Switzerland [1]. Providing an overall assessment of the nutritional quality of foods and beverages using a summary graded coloured system (from A, green for healthier products, to E, dark orange for less healthy products), the Nutri-Score was initially developed by an academic independent research group. The Nutri-Score was first introduced as the officially endorsed FOPL in France in 2017 after a three-year debate and following several comparative studies of its effectiveness on consumers’ purchasing behaviour [7,8,9,10,11,12]. It was registered as a brand owned by the public national Health Promotion Agency to ensure that companies adopting it follow the same standards for its computation and format (size and place on-pack). Its large voluntary implementation by manufacturers and retailers since its adoption in France in 2017—with more than 500 companies having registered for the system in 2021 [13]—and large awareness in the population [14] have led health authorities to consider its extension to other settings than pre-packed foods, including raw products or out-of-home eating [15].

In France, prior to the COVID-19 pandemic, 60.1% of adults were estimated to eat out of home at least once a week in 2014–2015, a percentage reaching 78.2% of the employed population [16]. Out-of-home eating represented an increasing household expenditure over time, from 14% of food household expenditure in 1959 to 26% in 2014 [17]. Growth in the sector of out-of-home eating was estimated at 2.8% for the year 2018, but with differing levels for catering (+1.8%) and commercial chain restaurants, including fast-food (+3.3%) [18]. While the COVID-19 pandemic drastically modified behaviours regarding out-of-home meals, severe activity restrictions pertaining to this sector did not expand beyond the first wave of the epidemic in the spring of 2020. Data on growth in the sector however need to be considered with caution.

Among French employed adults, around 20% usually frequent staff cafeterias at their place of work for lunch [16], representing several hundred million meals served every year [19]. Out-of-home catering provides a selection of products under a conventional structure of meals, including starters, main course and cheese or dessert, with a large variability in the nutritional quality of proposed products (e.g., starters may include salads and raw vegetables as well as processed meat). Given this variability, out-of-home catering in professional settings would provide an adequate environment for the introduction of nutrition labelling, which could foster healthier choices at the place of work [20,21].

Furthermore, investigating the impact of the Nutri-Score in catering would provide important insights on the effect of such a policy on food consumption and dietary intake. Indeed, while a number of experimental and field trials have investigated the effect of FOPLs—and Nutri-Score in particular—on food purchases in supermarkets [9,10,22,23,24], evidence on the impact of the implementation of front-of-pack nutrition labels on dietary intake is limited. Such insights would however be important to understand the potential for sustained dietary changes of FOPLs. Finally, considering the large share of the population regularly frequenting staff cafeterias in France, the implementation of the Nutri-Score in such an environment could lead to substantial gains, should it prove efficient to improve dietary intakes.

Our objective was to investigate the impact of the Nutri-Score on the nutritional quality of meals and dietary intakes of individuals frequenting staff cafeterias before and after implementation, using a quasi-experimental design. A secondary objective was to investigate the effects on dietary intakes over time during the experiment.

## 2. Materials and Methods

### 2.1. Design

The trial follows a quasi-experimental design, comparing food choices of participants frequenting a control or intervention staff restaurant run by the ELIOR group (catering company). Detailed data on food choices were collected daily, including a period before and a period after the implementation of the intervention, allowing for a double comparison before/after and control/intervention groups. Participants’ choices were recorded through their badge, used routinely for billing meals. Sites of similar size were selected from the same company and with matching food offer. Both restaurants were located in the large Paris area. In accordance with ethics and regulation rules, participants were informed that anonymised data on their food choices were collected over the study period, and had the option requesting their data to be removed from the analyses. The information regarding the study was present at the register/cashier at the end of the line, after participants had already made their food choices.

The trial was approved by the Inserm Institutional Review Board (approval number n°19-610) and was registered with the Commission Nationale de l’Informatique et des Libertés (CNIL registration number 2213956 v0). The trial was registered with clinicaltrials.gov (accessed on 5 December 2021) (identifier NCT04252898).

### 2.2. Data Collection

Detailed data on food choices for lunch were collected through receipts from the staff restaurants from 16 December 2019 to 13 March 2020, for a total of 13 weeks. The study was initially planned to proceed up to June 2020, but the general lockdown which commenced in March 2020 in response to the COVID-19 pandemic prematurely ended the trial.

All employees frequenting the staff restaurants were invited to participate (i.e., estimated at more than 300 meals served a day on average during the year). Participants were assigned anonymous numbers, allowing to follow their food selection throughout the study based on their personal badge, which is routinely used for billing meals. No socio-demographical data were collected. No incentives to participate were integrated in the trial.

Participants in staff restaurants were offered a selection of starters (ten starters, including salad bar), main courses (eight main per day), cheese (from a selection of various cheeses) and desserts (seven per day, including dessert bar, fruits and a selection of various yogurts), as well as several beverages. A specific selection was proposed each day as a ‘menu’ at a reduced price, including two options regarding starters, main courses and cheese or desserts. The food offer for the company was elaborated on a quarterly basis with a rotation of the various elements in the menus (starters, main courses and desserts) every month. Receipts contained details of the food choice selection, except for ‘cheese’, ‘yogurt’, ‘beverage’, ‘salad bar’, ‘desserts bar’, ‘side dishes’ or ‘menu’, which were not detailed.

### 2.3. Intervention

The intervention consisted of the implementation of the Nutri-Score label on all foods and non-alcoholic beverages served in the intervention staff restaurant, starting 3 February 2020 following a 7-week control period from 16 December.

Briefly, the Nutri-Score is a summary, graded and coloured FOPL, providing an overall assessment of the nutritional quality of a food, based on its nutritional composition. The Nutri-Score for a selection of foods is presented in Supplemental Table S1. Nutrients and elements considered—for 100 g or mL—are energy density, saturated fats, sugars, salt, proteins, fibres and percentage of fruit, vegetables, legumes, nuts and vegetable oils (canola, olive and nuts). The algorithm underpinning the Nutri-Score is the Food Standards Agency nutrient profiling system, modified for the purpose of food labelling by the French High Council for Public Health (FSAm/HCSP, detailed information on the computational algorithm in Supplemental Figure S1). The nutrient profile has been validated extensively against food composition, dietary behaviour and association with health events [25,26,27,28,29,30,31]. By construction, the higher the FSAm/HCSP, the lower the overall nutritional quality of the food or beverage.

All the products used as ingredients by the ELIOR group for the foods prepared in the staff restaurants were analysed for nutrient composition, either from national general databases (for raw ingredients) or from suppliers (for processed foods). Recipes were standardised (i.e., cooks were trained to strictly follow the recipe and cooks coordinated between intervention and control site to propose the same recipes each day) and the nutritional composition for the final products were calculated, considering the impact of cooking on the volume of individual ingredients (edible portion and yield following cooking). The calculation of the final composition of the recipes and the associated Nutri-Score were developed by ELIOR via their proprietary management software, providing the appropriate labels for all recipes. All foods in the ‘salad bar’ or ‘desserts bar’ and in the selection of cheese, yogurts and beverages were individually labelled, even though their detail did not appear in the receipts.

Data on the nutritional composition of products proposed directly to customers (e.g., beverages, yogurts, cheese) were either available in the back-of-pack nutrient declaration or retrieved from suppliers.

The Nutri-Score was affixed on the display labels of all foods and beverages in the restaurant alongside the price. Additionally, the Nutri-Score was displayed on the menus at the entrance of the restaurant. Finally, a communication campaign accompanied the introduction of the Nutri-Score, including poster displays at various locations in the restaurant and informational displays presenting the Nutri-Score, its computation and the way to use it on all tables. During the first days of the intervention, researchers were present on location to present the Nutri-Score and its use and respond to any queries by participants. Considering the large awareness of the Nutri-Score in the population in 2019 [14], no additional communication material was presented to participants.

A communication campaign of similar magnitude (i.e., posters and table informational displays) was displayed in the control restaurant, but regarding physical activity. The setting of the communication campaign for the control site aimed at standardising the type and magnitude of interventions between the sites except for the display of the label on foods and beverages.

A complementary web-based voluntary survey was sent to employees on both sites with invitation via email after the interruption of the trial to investigate their perception of the intervention. However, given that the national lockdown imposed an end to the trial, with all employees working remotely from 13 March 2020, the circumstances of administration of the survey were somewhat disconnected from their actual experiences in staff restaurants.

### 2.4. Statistical Analysis

Receipts included all the products consumed at a single meal by each participant, with one receipt per meal. Products on the receipts were matched with their corresponding nutritional composition, detailing individual nutrients (energy, carbohydrates, sugars, fats, saturated fats, proteins, salt and fibres) and the average portion size served. Portion sizes were specific for each recipe. For products that were proposed in the salad bar or dessert bar, the portion size was standardised based on the container used (e.g., small, medium, large). Standardised portions were used for cheese (i.e., 30 g) and fruit, and for yogurts and beverages the standard container was used. For side dishes, ‘beverages’, ‘cheese’, ‘yogurt’, ‘salad bar’ and ‘dessert bar’, an average composition of the foods proposed on the given day were computed. For ‘menus’, given the variability in the nutritional composition of the selection each given day, we elected not to compute average composition and exclude those meals from the analyses.

Food choices were weighted by the portion size (i.e., the nutrient content per 100 g from the nutritional composition table was multiplied by the portion size for each food) and summed for each day when the participant frequented the staff restaurant in order to obtain the total nutrient intake over the meal. The average nutritional quality of the meal was assessed using the algorithm underpinning the Nutri-Score, namely the FSAm/HCSP, averaged over the products selected for each meal and weighted according to the energy provided by each product, taking into account portion size.

If a receipt contained more than one main course or more than a total of 10 products, the corresponding receipts were excluded from the analysis, considering that they corresponded to the meal of more than one person on the same receipt. As no nutrient composition for the daily ‘menus’ were computable, those receipts were excluded. The distribution of energy intake over the meals were analysed, and meals with energy inferior to the 1st percentile (139 Kcal) or over the 99th percentile (1660 Kcal) of the distribution were excluded from the analyses.

Descriptive analyses on the participating staff restaurants were computed according to control or intervention site (total number of meals served over the period, average number of meals per day, average number of products per meal, total number of participants included and average number of meals participants took at the staff restaurant). Average nutrient intake and nutritional quality of meals over the study periods (before/after intervention) according to the control or intervention site were described using mean +/− SD for the nutritional quality of meals (using the energy-weighted FSAm/HCSP of the foods composing the meal) and each nutrient. Comparisons between before and after the intervention and the intervention and control sites for participants having at least one meal in each of the study periods were investigated using a difference in difference approach using unadjusted generalised linear models [32].

The impact of the intervention on the overall nutritional quality of the meals choices and nutrient intakes was investigated using mixed effects models with random intercepts in order to assess individual trajectories. The models included random individual intercept (accounting for varying individual baseline values), fixed effects for time (continuous, accounting for an overall seasonal trend in food choices), site (as 0 for the control site and 1 for the intervention site—accounting for differences in food choices between control and intervention sites throughout the study period) and interaction between time and site (site × time, accounting for differing seasonal trends in food choices over time between control and intervention sites). The effect of the intervention was modelled as a fixed effect for the intervention period (as 0 for the control site for the before and after periods, 0 for the intervention site for the before period and 1 for the intervention site for the after period—accounting for an immediate effect of the intervention) and an interaction between time and the intervention period (intervention period × time accounting for a modified trend over time after the intervention started). Trends modelled are presented graphically in Supplemental Figure S2.

Models were adjusted on the average nutritional quality of the offer on a given day as a fixed effect, in order to account for the day-to-day changes in the proposed offer. Figures of observed and predicted values were developed to provide a graphical visualisation of the results of the models. Sensitivity analyses with no adjustment on the average nutritional quality of each day and including only participants with at least 5 meals over the study (with at least one in each study period).

A priori power calculations with effect size set at 0.20, 0.90 power and 0.05 alpha risk showed that 1053 subjects would be required to be included, based on effects size estimates obtained from experimental studies performed on purchasing intentions [11] or in catering environments [33].

All tests were two-sided and a *p* value < 0.05 was considered significant. Statistical analyses were performed using SAS Software (version 9.3, SAS Institute Inc., Cary, NC, USA).

## 3. Results

**Figure 1:**
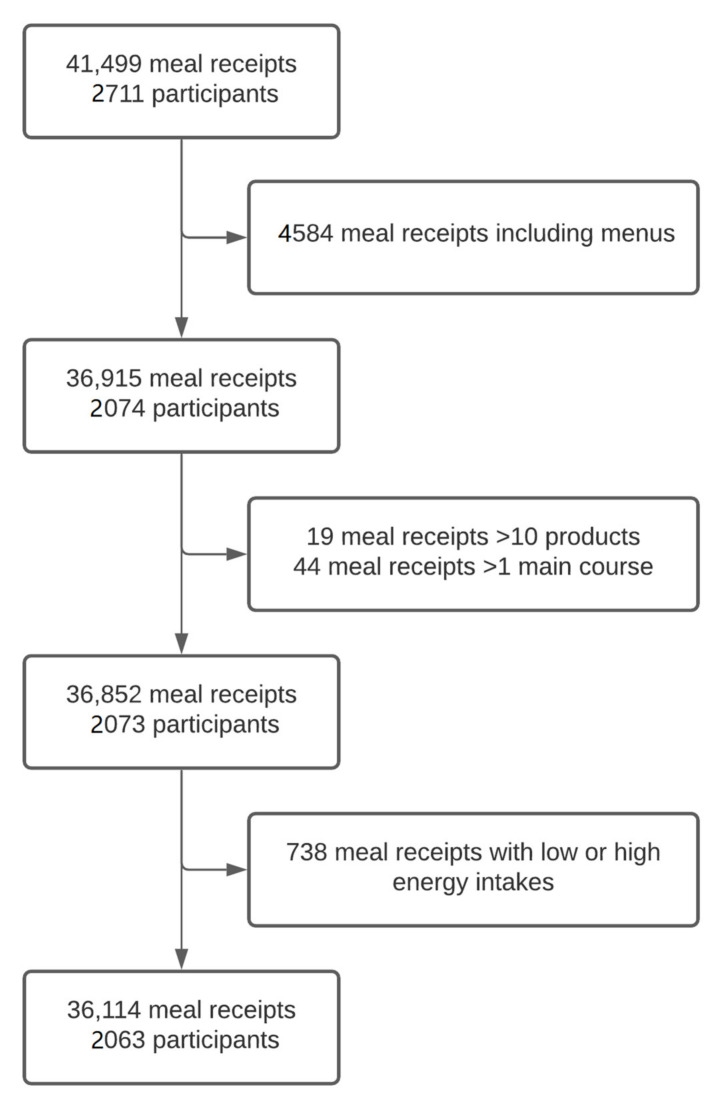
Flow chart of the inclusions in the analysis—Nutri-Score catering trial.

Between 16 December 2019 and 13 March 2020, 131,028 products were purchased by participants, corresponding to 41,499 meals served to 2711 participants over the study period (Figure 1). After excluding receipts including a menu (4584 meal receipts), those with more than 10 products (19 meal receipts) and receipts with more than one main course (44 meal receipts), 36,852 meals served to 2073 participants were included. Finally, after the exclusion of 738 meals with either extremely low or extremely high energy intake, a total of 36,114 meals (20,492 in the intervention site and 15,622 in the control site) served to 2063 (1268 and 795 in the intervention and control site, respectively—mean number of meals per participant 16.26 ± 13.54 and 19.65 ± 14.51, respectively) participants were included in the analyses (Figure 1).

The intervention site served more meals on average and in total than the control site, with an average of 330.52 ± 93.72 vs. 251.97 ± 65.53 meals per day during the study period (Table 1).

Before the intervention start, mean FSAm/HCSP score for the meal was 1.91 ± 2.31 for the intervention site and 2.03 ± 2.21 for the control site. After the intervention start, the mean was reduced to 1.69 ± 2.20 (corresponding to an improvement in the nutritional quality of meals) while it was 1.97 ± 2.00 in the control site (Table 2). Difference in difference models including participants having at least one meal in each study period showed similar results with statistically significant differences (*p* = 0.008, Supplemental Table S2).

Quantitatively, energy per meal was reduced in the intervention site from 811.67 ± 174.73 KCal/meal to 747.15 ± 169.80 KCal/meal, while it was modified from 851.37 ± 183.32 Kcal/meal to 831.91 ± 173.96 Kcal/meal in the control site. Overall, all nutrient intake displayed a decrease between the before/after period and intervention and control sites (Table 2). Difference in difference models in participants having at least one meal in each study period showed similar results, with all differences being statistically significant, except for salt and proteins (*p* < 0.0001 for energy, saturated fat and sugar, *p* < 0.02 for fibers, Supplemental Table S2).

**Figure 2:**
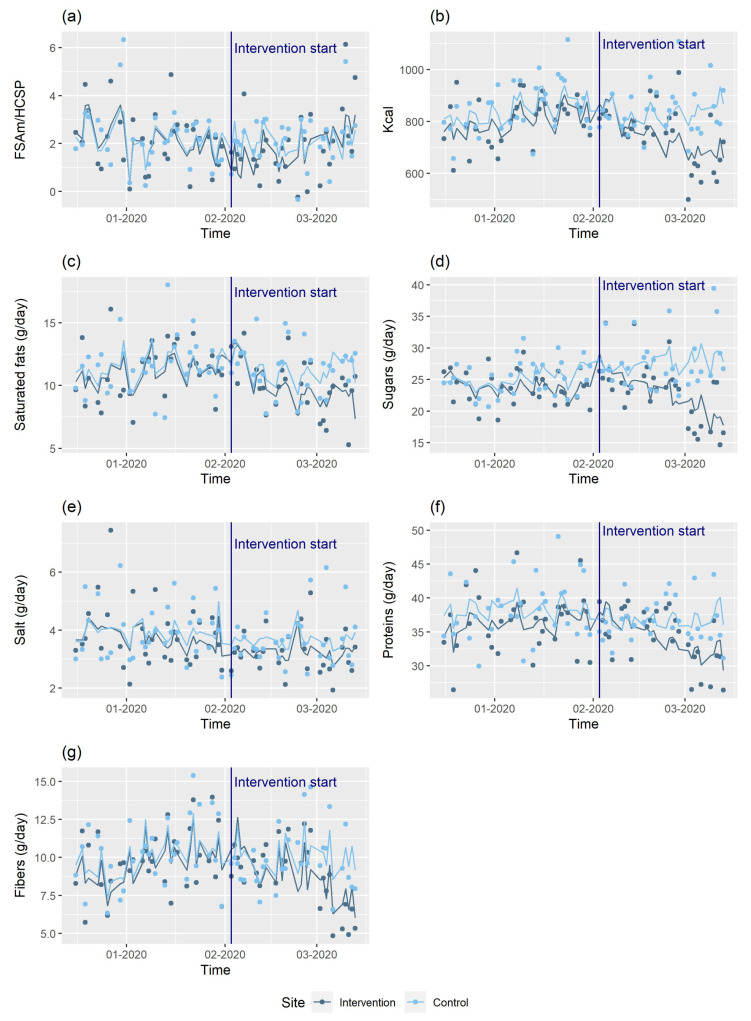
Observed and predicted dietary intakes over the study period for the intervention and control sites—Nutri-Score catering trial. (**a**) FSAm/HCSP score; (**b**) energy (Kcal/day); (**c**) saturated fats (g/day); (**d**) sugars (g/day); (**e**) salt (g/day); (**f**) proteins (g/day); (**g**) fibers (g/day).

Mixed effects models showed the intervention had a significant immediate beneficial impact on the overall nutritional quality of meals (βFSAm/HCSP for intervention period −3.37, 95% confidence interval (95% CI): −3.78; −2.95), *p* < 0.0001, a lower FSAm/HCSP corresponding to higher nutritional quality), though this favourable effect tended to subside over time (βFSAm/HCSP for intervention period × time 0.052 (0.045; 0.06), *p* < 0.0001) (Table 3, Figure 2). Quantitatively, the intervention had a significant immediate impact on nutrient intake, with increases in calories and all nutrients except salt, but with a subsequent significant decrease over time which superseded this immediate effect (Table 3, Figure 2). For salt, the opposite trends were observed, with an immediate significant decrease followed by a significant increase over time (Table 3, Figure 2). Sensitivity analyses yielded similar results to the main analyses (Supplemental Tables S3 and S4).

Following the trial termination, 72 participants responded to the online survey. Among them, a majority declared having noticed and seen the communication material pertaining to the Nutri-Score (64.2% positive responses overall), mostly the tags on the shelf (76.7% of participants having noticed the Nutri-Score). Communication on the menus (46.5%) or on the tables (37.2%) were less frequently noticed (Supplemental Table S5).

## 4. Discussion

The results of our study show that the introduction of the Nutri-Score in real-life collective catering conditions leads to qualitative and quantitative modifications of food choices, with improvements in the nutritional quality of meals (i.e., FSAm/HCSP, as the nutrient profile is computed on 100 g of product) and the amounts of unfavourable nutrients intake. Our results also showed that while the qualitative effect on the nutritional quality of meals was immediate and not necessarily lasting over time, quantitative effects on nutrient intake were initially unfavourable but with a significant improvement over time (except fibres), suggesting adaptations in the quantities or portion sizes consumed in the longer term.

Previous studies have shown positive effects of the Nutri-Score on food purchases, either in experimental or real-life conditions [9,10,22,23]. Short-term studies in experimental settings in particular have shown the Nutri-Score to reduce purchases of calories, sugars and saturated fats [22,23], and increase in purchases of fruit and vegetables [22]. A large scale medium-term trial over five weeks conducted in French supermarkets showed that the Nutri-Score led to improvements in the overall nutritional quality of purchases, though no details were provided as to the specific effects on nutrient contents of the shopping cart [10]. These results are in line with a recent meta-analysis exploring the impact of front-of-pack labels on objectively measured consumption [4], showing lower purchases of sodium and sugar in front-of-pack labelling conditions. However, only a very limited number of studies included in the review reported effects on consumption (*n* = 3), they were all experimental and none investigated the Nutri-Score [4]. Our results therefore expand previous research by exploring the effects of the introduction of the Nutri-Score on food consumption and dietary intake in a pragmatic trial.

While the overall nutritional quality of meals was initially improved (effects observed on the FSAm/HCSP of meals), quantitatively participants initially consumed higher amounts of unfavourable nutrients, suggesting the Nutri-Score may have had an initial halo effect, with consumption of higher amounts of ‘healthier’ products [34]. Increase in sugar intake in particular could be due to an increase in the consumption of fruits, which has been observed in trials investigating purchasing intentions [22]. However, this initial effect was followed by a significant reduction in the intake of all nutrients, suggesting an adaptation of the quantities consumed with lower portions sizes selected over time. Previous experimental data suggest that the implementation of a Nutri-Score D or E on less healthy products may lead to lower portion selection [35]. Considering that the food offered in the staff cafeterias covered a wide range of foods of various nutritional value, our results would tend to suggest that the effects of the Nutri-Score on portion size selection may extend beyond solely those with lower nutritional value. This hypothesis would however need to be confirmed by longer-term studies in this type of environment.

Results in other settings have shown inconsistent effects of labelling in out-of-home eating environments, the most widely studied intervention being calorie labelling, which has been mandated in some countries [36,37]. Restaurant menu calorie labelling has shown a limited impact on calories consumed, with a high heterogeneity between studies [37]. Heterogeneity appeared to be linked to the type of setting involved, with more positive results in settings like coffee shops, some fast-food restaurants (e.g., sandwich shops) or full-service restaurants [38]. A recent pilot study investigating the impact of calorie labelling in worksite cafeterias in the UK showed non-significant effects of the intervention [39]. Conversely, a large quasi-experimental study conducted in a large fast-food chain in the US found a significant effect of calorie menu labelling on the calories purchased by transaction [40]. However, the initial effect observed on calories purchased appeared to decrease over time, while in our study, though the impact of the Nutri-Score on the quality of the content of meals did indeed decrease over time, the opposite was observed for the quantity of calories consumed. In line with our results, a meta-analysis of 38 studies covering a wide range of labelling interventions found that studies with positive results (either partial or overall) tended to be those conducted in cafeterias and providing interpretive information (in the form of ‘positive’ or traffic light labels) [41].

Beyond their effect on food choices, front-of-pack labels are thought to act as incentives for modification in the food offer, though results in this area tend to be highly variable [42]. The introduction of calorie labelling in Canada did not appear to lead to modifications in the food offer, with even an increase in serving sizes and mean calories between 2010 and 2017 [43]. Given the quasi-experimental design of our study, we required no modifications in recipes during the study period to be made. Indeed, this allowed us to ensure that the impact observed was due to modifications in food choices and not to modifications in the nutritional composition of the food offer. However, after completion of the study, ELIOR started revising recipes to improve the Nutri-Score, showing the incentive provided by the implementation of the measure and a potential for improvement in dietary intake through reformulation in the future.

Strengths of our study include its quasi-experimental design allowing to account for seasonal trends in food choices and baseline differences between the intervention and control sites. Moreover, we were able to investigate the effects of the intervention over time with individual trajectories in food choices, providing insights into the mid-term effect of the introduction of a front-of-pack labelling. Finally, the design allowed to investigate the effects of the Nutri-Score on nutrient intake in real-life conditions, as foods selected in a catering environment are directly consumed.

Our study is subject to several limitations. First, though we selected the intervention and control sites from the same company and with a similar background of employees, we cannot exclude that some of the differences observed may not be attributable to the intervention. However, we took into account potential differences between the sites in the various models both as baseline average effects and trends over time. We also mitigated observational bias by informing participants of the data collection after they had operated their first set of choices. Second, though menus and recipes were standardised between sites and restaurant staff were trained to follow the recipes in regards to contents in the various ingredients (salt and fat in particular), we cannot exclude that in some cases the actual composition of the foods may vary from the nutritional composition retained in the analyses. This limitation was identified in other studies investigating the impact of labelling in catering, as cooks have some freedom in adding salt or fat during cooking [39]. However, all chefs and cooks were trained before the study to ensure that both sites offered the same menus and that cooks would strictly follow recipes, and all restaurant teams favourably received the participation in the study. Moreover, this bias probably affected both the intervention and the control site, leading to a non-differential bias. Third, the lack of detail in some of the receipts did not allow us to take into account the detail of the individual choices that were made. In some cases, the high variability in the food offer for these elements led us to exclude the data (i.e., menus, leading to the exclusion of 637 participants), in other cases, the homogeneity in the food offer they represented (e.g., yogurts, cheese or salad bar with a relatively similar food composition) led us to maintain them with an average composition in the analysis. This could have led to a classification bias, albeit non-differential as it affected both the intervention and control sites. However, as menus costs were rather low budget, we may have excluded more vulnerable groups from the analysis. Fourth, we did not collect information as to the food waste and hence the actual quantity of food consumed by each individual, only purchase information was accessible through receipts. Again, this would be considered as a non-differential classification bias in the analysis. Furthermore, we were not able to have access to individual socio-demographic data, hence we were not able to take individual profiles in the models. We cannot rule out entirely the possibility that some participants may have used multiple badges and therefore be identified as multiple participants. However, the use of personal badges routinely applied for billing as a basis for individual identification minimised this bias. In addition, we excluded anonymised numbers that appeared in both intervention and control sites. Finally, our study was cut short by the COVID-19 pandemic and the government’s decision for a protracted lockdown initiating in March 2020, limiting our ability to take into account effects of the Nutri-Score over longer periods of time. However, we were able to investigate the impact of the Nutri-Score in the medium term, with six weeks follow-up.

## 5. Conclusions

Overall, our results indicate that the introduction of the Nutri-Score leads to favourable modifications in nutrient intake, with an initial improvement of the overall nutritional quality of meals and a longer-term quantitative effect with a reduction in quantities consumed, suggesting adaptating dietary behaviours in consumers over time. Future studies should explore further longer-term trends in the impact of front-of-pack labelling on food consumption to confirm these results, but these initial results suggest Nutri-Score would be useful to guide consumer choices in catering, confirming its effect beyond purchases in retail environments.

## Figures and Tables

**Table 1:** Descriptive analysis of the intervention and control sites—Nutri-Score catering trial.

	Intervention Site	Control Site
Total number of meals	20,492	15,622
Average number of meals per day	330.52 ± 93.72	251.97 ± 65.53
Average number of products per meal	3.32 ± 1.13	3.47 ± 1.07
Total number of participants	1268	795
Average number of meals per participant	16.16 ± 13.54	19.65 ± 14.51

Numbers are mean ± SD.

**Table 2:** Mean nutritional quality of means and nutrient intake per meal according to the period of the study and intervention and control sites in the Nutri-Score catering trial.

	Intervention Site		Control Site	
	Before	After	Before	After
N	1119	1100	728	710
FSAm/HCSP of the meal ^1^	1.91 ± 2.31	1.69 ± 2.20	2.03 ± 2.21	1.97 ± 2.00
Energy (Kcal/meal)	811.67 ± 174.73	747.15 ± 169.80	851.37 ± 183.32	831.91 ± 173.96
Sugars (g/meal)	24.03 ± 10.24	23.45 ± 10.28	25.55 ± 11.70	27.83 ± 12.04
Saturated fat (g/meal)	11.21 ± 4.37	10.00 ± 3.78	11.51 ± 4.24	11.29 ± 3.78
Salt (g/meal)	3.65 ± 1.38	3.25 ± 1.17	3.96 ± 1.54	3.55 ± 1.17
Proteins (g/meal)	36.08 ± 10.48	34.26 ± 8.72	38.53 ± 10.07	36.82 ± 9.08
Fibers (g/meal)	10.13 ± 3.26	9.14 ± 3.06	10.58 ± 3.02	9.95 ± 2.56

Numbers are mean ± SD. ^1^ FSAm/HCSP is the nutrient profiling model underpinning the Nutri-Score and corresponds to the overall nutritional quality of the meal (qualitative assessment). It was calculated using the energy-weighted mean of foods composing each meal.

**Table 3:** Impact of the Nutri-Score on the overall nutritional quality of meals and nutrient intake over time—Nutri-Score catering trial.

		β	CI 95%	*p*
FSAm/HCSP ^1^ of the meal				
	Site	0.33	0.08; 0.58	0.009
	Time	0.006	0.004; 0.008	<0.0001
	Time × site	−0.013	−0.018; −0.007	<0.0001
	Intervention period	−3.37	−3.78; −2.95	<0.0001
	Intervention period × time	0.052	0.045; 0.06	<0.0001
Calories (g/meal)				
	Site	−39.91	−58.82; −20.99	<0.0001
	Time	1.01	0.84; 1.17	<0.0001
	Time × site	0.14	−0.25; 0.52	0.50
	Intervention period	353.53	325.44; 381.62	<0.0001
	Intervention period × time	−5.95	−6.46; −5.44	<0.0001
Sugars (g/meal)				
	Site	0.85	−0.27; 1.97	0.14
	Time	0.03	0.02; 0.04	<0.0001
	Time × site	−0.08	−0.1; −0.06	<0.0001
	Intervention period	14.74	13.16; 16.32	<0.0001
	Intervention period × time	−0.21	−0.24; −0.18	<0.0001
Saturated fats (g/meal)				
	Site	−0.41	−0.86; 0.04	0.07
	Time	0.017	0.013; 0.021	<0.0001
	Time × site	0.004	−0.006; 0.014	0.42
	Intervention period	4.35	3.63; 5.07	<0.0001
	Intervention period × time	−0.08	−0.09; −0.07	<0.0001
Salt (g/meal)				
	Site	0.16	0.01; 0.31	0.04
	Time	0.002	0.001; 0.003	0.005
	Time × site	−0.013	−0.016; −0.009	<0.0001
	Intervention period	−0.42	−0.67; −0.16	0.001
	Intervention period × time	0.01	0.01; 0.02	<0.0001
Proteins (g/meal)				
	Site	−3.13	−4.15; −2.11	<0.0001
	Time	0.02	0.01; 0.03	0.0001
	Time × site	0.04	0.02; 0.06	0.0002
	Intervention period	14.16	12.66; 15.67	<0.0001
	Intervention period × time	−0.24	−0.27; −0.21	<0.0001
Fibres (g/meal)				
	Site	−0.6	−0.91; −0.29	0.0002
	Time	0.001	−0.002; 0.003	0.59
	Time × site	0.006	0; 0.013	0.06
	Intervention period	5.92	5.44; 6.4	<0.0001
	Intervention period × time	−0.1	−0.11; −0.09	<0.0001

β obtained from mixed effects models, adjusted for the average nutrient composition for each day. The models included fixed effects for time (accounting for an overall seasonal trend in food choices), site (accounting for differences in food choices between control and intervention sites throughout the study period) and interaction between time and site (site × time, accounting for differing seasonal trends in food choices over time between control and intervention sites). The effect of the intervention was modelled as a fixed effect for the intervention period (accounting for an immediate effect of the intervention) and an interaction between time and the intervention period (intervention period × time accounting for a modified trend over time after the intervention started). ^1^ FSAm/HCSP is the nutrient profiling model underpinning the Nutri-Score and corresponds to the overall nutritional quality of the meal (qualitative assessment). It was calculated using the energy-weighted mean of foods composing each meal.

## Data Availability

The data supporting the conclusion of the manuscript are available upon request and review from the Nutri-Score catering trial team.

## Supplementary Materials

The following are available online at https://www.mdpi.com/article/10.3390/nu13124530/s1, Figure S1: Point attribution and allocation to the Nutri-Score based on the nutritional composition of the food, Figure S2: Graphical representation of the effects modelled in the main analysis—mixed effects models, Table S1: Nutri-Score associated with a number of food products from the Nutri-Score catering trial, Table S2: Mean nutritional quality of means and nutrient intakes per meal according to the period of the study and intervention and control sites in the Nutri-Score catering trial—difference in difference analysis for participants with at least one meal in each study period, Table S3: Impact of the Nutri-Score on the overall nutritional quality of meals and nutrient intakes over time—sensitivity analysis with no adjustment on the average nutrient composition of the daily offer. Nutri-Score catering trial, Table S4: Impact of the Nutri-Score on the overall nutritional quality of meals and nutrient intakes over time—sensitivity analysis including only participants with more than five meals over the study (at least one meal in each study period). Nutri-Score catering trial (N = 1456), Table S5: Results from the complementary voluntary online survey administered after trial termination N = 72.
