# Supplementary material for: Impact of the Front-of-Pack Label Nutri-Score on the Nutritional Quality of Food Choices in a Quasi-Experimental Trial in Catering
Source: Nutrients. 2021 Dec 17;13(12):4530. doi: 10.3390/nu13124530 (PMC8706580; doi:10.3390/nu13124530)
Supplement: Supplementary file 1 [file nutrients-13-04530-s001.zip › nutrients-1480781-supplementary.pdf]

Table S1 Nutri-Score associated with a number of food products from the Nutri-Score catering trial

Food product	Nutri-Score
Ratatouille	NUTRI-SCORE A
Vegetables and mushroom risotto	NUTRI-SCORE B
Salmon tartar	NUTRI-SCORE B
Gratin Dauphinois	NUTRI-SCORE C
Crème caramel	NUTRI-SCORE C
Lemon mousse with biscuit	NUTRI-SCORE D
Chocolate cake	NUTRI-SCORE E

Figure S1 Point attribution and allocation to the Nutri-Score based on the nutritional composition of the food

1. Attribution of points. based on the content of nutrients and other elements per 100 g of a food/beverage

Points A

			Specific cut-offs: **Beverages**			Specific cut-offs: **Fats**	
**Points**	Energy (kJ)	Sugars (g)	Energy (kJ)	Sugars (g)	Saturated fat (g)	Saturated fat/Lipids (%)	Sodium (mg)
**0**	≤ 335	≤ 4.5	≤ 0	≤ 0	≤ 1	< 10	≤ 90
**1**	> 335	> 4.5	≤ 30	≤ 1.5	> 1	< 16	> 90
**2**	> 670	> 9	≤ 60	≤ 3	> 2	< 22	> 180
**3**	> 1005	> 13.5	≤ 90	≤ 4.5	> 3	< 28	> 270
**4**	> 1340	> 18	≤ 120	≤ 6	> 4	< 34	> 360
**5**	> 1675	> 22.5	≤ 150	≤ 7.5	> 5	< 40	> 450
**6**	> 2010	> 27	≤ 180	≤ 9	> 6	< 46	> 540
**7**	> 2345	> 31	≤ 210	≤ 10.5	> 7	< 52	> 630
**8**	> 2680	> 36	≤ 240	≤ 12	> 8	< 58	> 720
**9**	> 3015	> 40	≤ 270	≤ 13.5	> 9	< 64	> 810
**10**	> 3350	> 45	> 270	> 13.5	> 10	≥ 64	> 900
	**0-10 (a)**	**0-10 (b)**	**0-10 (a)**	**0-10 (b)**	**0-10 (c)**	**0-10 (c)**	**0-10 (d)**
**Total**	**Points A = (a) + (b) + (c) + (d) [0 – 40]**						

Points C

		Specific cut-offs: **Beverages**		
**Points**	Fruits. veg (%)	Fruits. veg (%)	Fiber (g)	Protein (g)
**0**	≤ 40	≤ 40	≤ 0.9	≤ 1.6
**1**	> 40		> 0.9	> 1.6
**2**	> 60	>40	> 1.9	> 3.2
**3**	-		> 2.8	> 4.8
**4**	-	> 60	> 3.7	> 6.4
**5**	> 80		> 4.7	> 8.0
**6**				
**7**				
**8**				
**9**				
**10**		> 80		
	**0-5 (a)**	**0-10 (a)**	**0-5 (b)**	**0-5 (c)**
**Total**	**Points C = (a) + (b) + (c) [0 – 15]**			

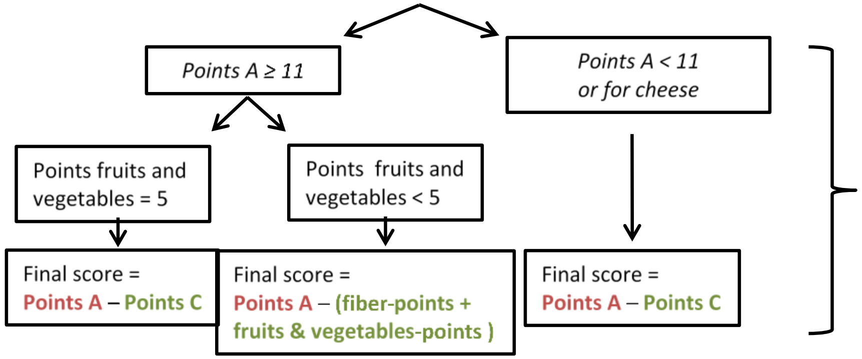

2. Final score: -15 to 40 points.

3. Attribution of colors:

Foods (points)	Beverages (points)	Color
Min to -1	Water	Dark green
0 to 2	Min to 1	Light green
3 to 10	2 to 5	Yellow
11 to 18	6 to 9	Light Orange
19 to Max	10 to Max	Dark orange

NUTRI-SCORE

Green: highest quality *Red: lowest quality*

Figure S2 Graphical representation of the effects modelled in the main analysis – mixed effects models

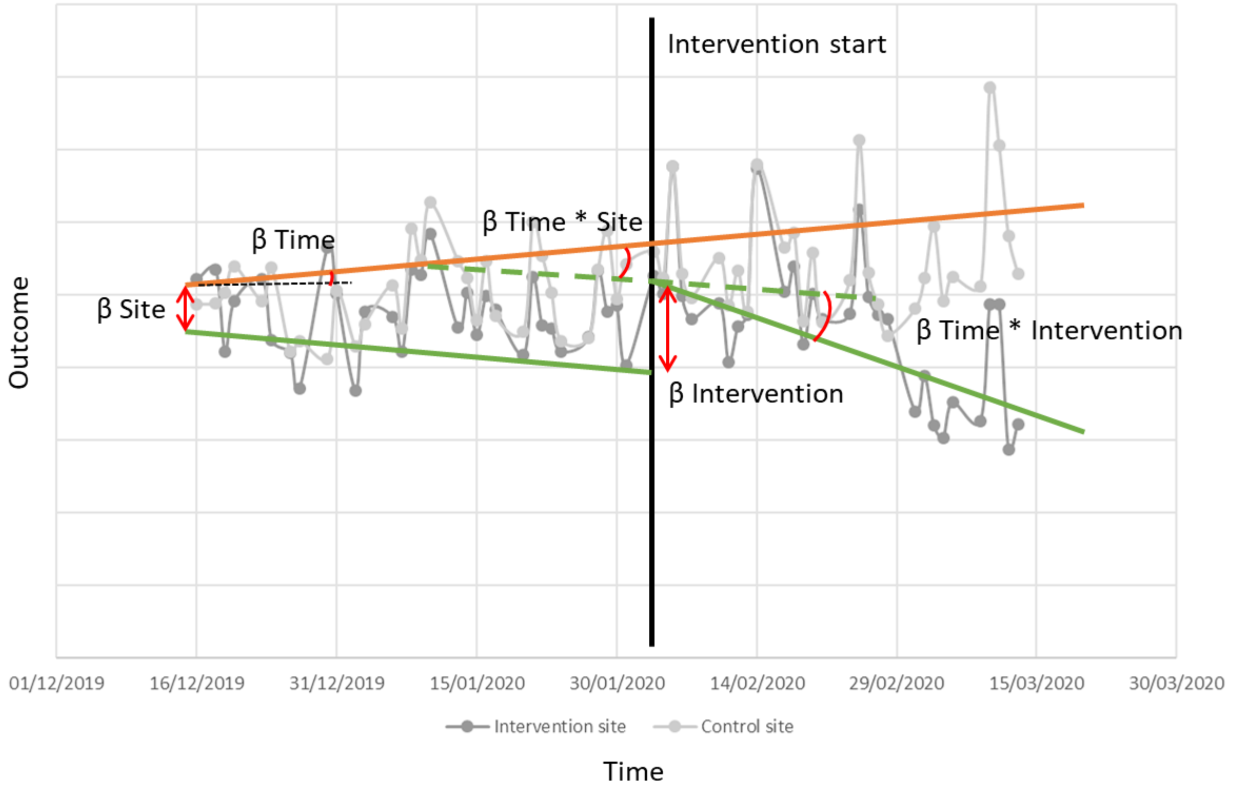

Table S2 Mean nutritional quality of means and nutrient intakes per meal according to the period of the study and intervention and control sites in the Nutri-Score catering trial – difference in difference analysis for participants with at least one meal in each study period

	**Intervention site**		**Control site**		***Difference before/after intervention site***	***Difference before/after control site***	***p DID*[2]**
	**Before**	**After**	**Before**	**After**			
N	951	951	643	643			
FSAm/HCSP of the meal[1]	1.95 ± 2.14	1.71 ± 2.06	1.98 ± 2.02	2.02 ± 1.90	*0.001*	*0.57*	0.008
Energy (Kcal/meal)	815.40 ± 163.97	747.87 ± 158.17	852.23 ± 175.16	834.14 ± 170.30	*<0.0001*	*0.0004*	<0.0001
Sugars (g/meal)	23.83 ± 9.59	23.39 ± 9.34	25.56 ± 11.03	27.88 ± 11.68	*0.12*	*<0.0001*	<0.0001
Saturated fat (g/meal)	11.26 ± 4.13	10.01 ± 3.51	11.50 ± 3.98	11.35 ± 3.69	*<0.0001*	*0.23*	<0.0001
Salt (g/meal)	3.70 ± 1.32	3.28 ± 1.10	3.92 ± 1.41	3.56 ± 1.06	*<0.0001*	*<0.0001*	0.48
Proteins (g/meal)	36.46 ± 9.73	34.32 ± 8.34	38.32 ± 9.80	36.79 ± 8.95	*<0.0001*	*<0.0001*	0.13
Fibers (g/meal)	10.13 ± 3.02	9.15 ± 2.83	10.61 ± 2.83	9.95 ± 2.42	*<0.0001*	*<0.0001*	0.02

Numbers are mean ±SD

[1]FSAm/HCSP is the nutrient profiling model underpinning the Nutri-Score and correspond to the overall nutritional quality of the meal (qualitative assessment). It was calculated using the energy-weighted mean of foods composing each meal

[2]Difference in difference analyses were carried out using unadjusted generalized linear models

Table S3 Impact of the Nutri-Score on the overall nutritional quality of meals and nutrient intakes over time – sensitivity analysis with no adjustment on the average nutrient composition of the daily offer. Nutri-Score catering trial

		β	IC 95%	*P*
**FSAm/HCSP[1] of the meal**				
	Site	0.46	0.21 ; 0.7	*0.0003*
	Time	0	-0.002 ; 0.002	*0.93*
	Time*site	-0.017	-0.023 ; -0.012	*<0.0001*
	Intervention period	-3.83	-4.25 ; -3.41	*<0.0001*
	Intervention period*time	0.06	0.05 ; 0.07	*<0.0001*
**Calories (g/meal)**				
	Site	-82.38	-101.31 ; -63.46	*<0.0001*
	Time	0.04	-0.11 ; 0.19	*0.60*
	Time*site	1.95	1.57 ; 2.33	*<0.0001*
	Intervention period	357.44	328.88 ; 385.99	*<0.0001*
	Intervention period*time	-7.35	-7.86 ; -6.84	*<0.0001*
**Sugars (g/meal)**				
	Site	0.69	-0.42 ; 1.81	*0.22*
	Time	0.054	0.046 ; 0.063	*<0.0001*
	Time*site	-0.08	-0.1 ; -0.06	*<0.0001*
	Intervention period	15.54	13.95 ; 17.12	*<0.0001*
	Intervention period*time	-0.22	-0.25 ; -0.19	*<0.0001*
**Saturated fats (g/meal)**				
	Site	-1.18	-1.63 ; -0.73	*<0.0001*
	Time	-0.003	-0.007 ; 0.001	*0.13*
	Time*site	0.03	0.02 ; 0.04	*<0.0001*
	Intervention period	6.13	5.4 ; 6.85	*<0.0001*
	Intervention period*time	-0.13	-0.14 ; -0.11	*<0.0001*
**Salt (g/meal)**				
	Site	0	-0.15 ; 0.15	*0.98*
	Time	-0.004	-0.005 ; -0.002	*<0.0001*
	Time*site	-0.006	-0.009 ; -0.002	*0.0009*
	Intervention period	-0.68	-0.94 ; -0.42	*<0.0001*
	Intervention period*time	0.01	0.005 ; 0.014	*<0.0001*
**Proteins (g/meal)**				
	Site	-3.92	-4.94 ; -2.9	*<0.0001*
	Time	-0.02	-0.03 ; -0.01	*<0.0001*
	Time*site	0.08	0.06 ; 0.1	*<0.0001*
	Intervention period	12.97	11.46 ; 14.47	*<0.0001*
	Intervention period*time	-0.25	-0.28 ; -0.23	*<0.0001*
**Fibres (g/meal)**				
	Site	-1.33	-1.65 ; -1.02	*<0.0001*
	Time	-0.002	-0.005 ; 0.001	*0.19*
	Time*site	0.04	0.03 ; 0.05	*<0.0001*
	Intervention period	6.15	5.65 ; 6.65	*<0.0001*
	Intervention period*time	-0.13	-0.13 ; -0.12	*<0.0001*

β obtained from mixed effects models.

The models included fixed effects for time (accounting for an overall seasonal trend in food choices), site (accounting for differences in food choices between control and intervention sites throughout the study period) and interaction between time and site (site*time, accounting for differing seasonal trends in food choices over time between control and intervention sites). The effect of the intervention was modelled as a fixed effect for the intervention period (accounting for an immediate effect of the intervention) and an interaction between time and the intervention period (intervention period*time accounting for a modified trend over time after the intervention started).

[1]FSAm/HCSP is the nutrient profiling model underpinning the Nutri-Score and correspond to the overall nutritional quality of the meal (qualitative assessment)

Table S4 Impact of the Nutri-Score on the overall nutritional quality of meals and nutrient intakes over time – sensitivity analysis including only participants with more than five meals over the study (at least one meal in each study period). Nutri-Score catering trial (N=1456)

		β	IC 95%	*P*
**FSAm/HCSP[1] of the meal**				
	Site	0.36	0.1 ; 0.61	*0.007*
	Time	0.006	0.004 ; 0.008	*<0.0001*
	Time*site	-0.012	-0.018 ; -0.007	*<0.0001*
	Intervention period	-3.34	-3.76 ; -2.91	*<0.0001*
	Intervention period*time	0.05	0.04 ; 0.06	*<0.0001*
**Calories (g/meal)**				
	Site	-41.89	-62.02 ; -21.77	*<0.0001*
	Time	1.02	0.86 ; 1.19	*<0.0001*
	Time*site	0.2	-0.19 ; 0.6	*0.31*
	Intervention period	355.22	326.49 ; 383.94	*<0.0001*
	Intervention period*time	-6.03	-6.55 ; -5.51	*<0.0001*
**Sugars (g/meal)**				
	Site	0.68	-0.51 ; 1.87	*0.27*
	Time	0.03	0.02 ; 0.04	*<0.0001*
	Time*site	-0.08	-0.1 ; -0.06	*<0.0001*
	Intervention period	14.54	12.93 ; 16.15	*<0.0001*
	Intervention period*time	-0.21	-0.23 ; -0.18	*<0.0001*
**Saturated fats (g/meal)**				
	Site	-0.4	-0.87 ; 0.08	*0.10*
	Time	0.017	0.013 ; 0.021	*<0.0001*
	Time*site	0.004	-0.006 ; 0.014	*0.41*
	Intervention period	4.46	3.71 ; 5.2	*<0.0001*
	Intervention period*time	-0.08	-0.1 ; -0.07	*<0.0001*
**Salt (g/meal)**				
	Site	0.19	0.03 ; 0.34	*0.02*
	Time	0.002	0.001 ; 0.004	*0.002*
	Time*site	-0.013	-0.016 ; -0.009	*<0.0001*
	Intervention period	-0.4	-0.66 ; -0.14	*0.002*
	Intervention period*time	0.011	0.006 ; 0.016	*<0.0001*
**Proteins (g/meal)**				
	Site	-3.01	-4.09 ; -1.92	*<0.0001*
	Time	0.02	0.01 ; 0.03	*0.0001*
	Time*site	0.04	0.02 ; 0.06	*0.0001*
	Intervention period	14.27	12.73 ; 15.8	*<0.0001*
	Intervention period*time	-0.24	-0.27 ; -0.22	*<0.0001*
**Fibres (g/meal)**				
	Site	-0.58	-0.9 ; -0.25	*0.0006*
	Time	0.001	-0.002 ; 0.003	*0.55*
	Time*site	0.006	-0.001 ; 0.012	*0.11*
	Intervention period	5.85	5.36 ; 6.35	*<0.0001*
	Intervention period*time	-0.096	-0.105 ; -0.087	*<0.0001*

β obtained from mixed effects models, adjusted for the average nutrient composition for each day. The models included fixed effects for time (accounting for an overall seasonal trend in food choices), site (accounting for differences in food choices between control and intervention sites throughout the study period) and interaction between time and site (site*time, accounting for differing seasonal trends in food choices over time between control and intervention sites). The effect of the intervention was modelled as a fixed effect for the intervention period (accounting for an immediate effect of the intervention) and an interaction between time and the intervention period (intervention period*time accounting for a modified trend over time after the intervention started).

[1]FSAm/HCSP is the nutrient profiling model underpinning the Nutri-Score and correspond to the overall nutritional quality of the meal (qualitative assessment)

Table S5 Results from the complementary voluntary online survey administered after trial termination. N=72

Are familiar with the Nutri-Score (have already heard or seen the Nutri-Score - unprompted) N=72		
Yes	57	79.2
No	10	13.9
Not sure	5	6.9
Have noticed the Nutri-Score during the study period N=67		
Yes	43	64.2
No	17	25.4
Not sure	7	10.4
Have noticed the Nutri-Score on (N=43)		
Shelf display	33	76.7
Menus	20	46.5
Information on tables	16	37.2
Displays in the restaurant (poster or screen displays)	9	20.9
Consider that Nutri-Score influenced their food choices N=67		
Yes, often	11	16.4
Yes, sometimes	29	43.3
Yes, a little	1	1.5
No	26	38.8
Already knew how to use Nutri-Score before it was implemented in the staff restaurant N=72		
Agree	56	77.8
Disagree	16	22.2
Would like for Nutri-Score to be maintained in the staff cafeteria N=72		
Agree	68	94.4
Disagree	4	5.6

Results presented are numbers and percentages
